# Leveraging Text-to-Text Pretrained Language Models for Question Answering in Chemistry

**DOI:** 10.1021/acsomega.3c08842

**Published:** 2024-03-12

**Authors:** Dan Tran, Laura Pascazio, Jethro Akroyd, Sebastian Mosbach, Markus Kraft

**Affiliations:** † CARES, Cambridge Centre for Advanced Research and Education in Singapore, 1 Create Way, CREATE Tower, #05-05, Singapore 138602, Singapore; ‡ Department of Chemical Engineering and Biotechnology, University of Cambridge, Philippa Fawcett Drive, Cambridge CB3 0AS, U.K.; § CMCL Innovations, Sheraton House, Castle Park, Cambridge CB3 0AX, U.K.; ∥ School of Chemical and Biomedical Engineering, Nanyang Technological University, 62 Nanyang Drive, Singapore 637459, Singapore; ⊥ The Alan Turing Institute, 96 Euston Rd., London NW1 2DB, U.K.

## Abstract

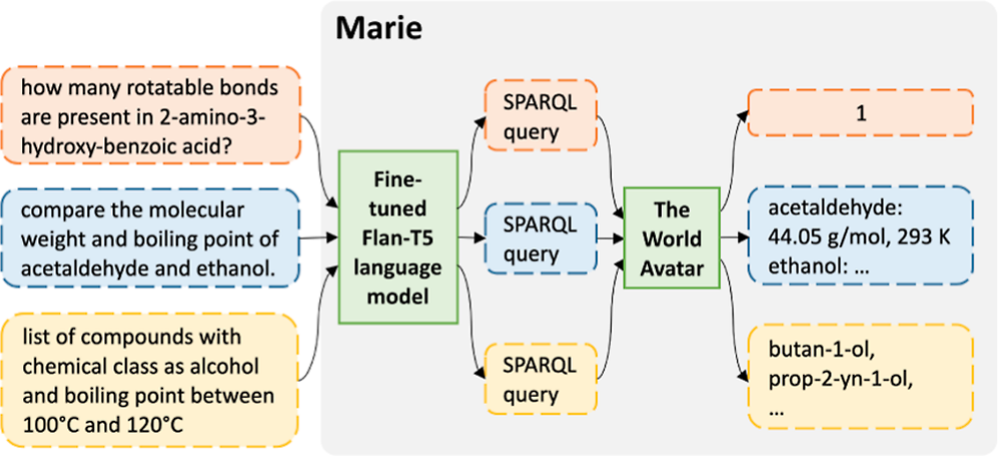

In this study, we present a question answering (QA) system for chemistry, named Marie, with the use of a text-to-text pretrained language model to attain accurate data retrieval. The underlying data store is “The World Avatar” (TWA), a general world model consisting of a knowledge graph that evolves over time. TWA includes information about chemical species such as their chemical and physical properties, applications, and chemical classifications. Building upon our previous work on KGQA for chemistry, this advanced version of Marie leverages a fine-tuned Flan-T5 model to seamlessly translate natural language questions into SPARQL queries with no separate components for entity and relation linking. The developed QA system demonstrates competence in providing accurate results for complex queries that involve many relation hops as well as showcasing the ability to balance correctness and speed for real-world usage. This new approach offers significant advantages over the prior implementation that relied on knowledge graph embedding. Specifically, the updated system boasts high accuracy and great flexibility in accommodating changes and evolution of the data stored in the knowledge graph without necessitating retraining. Our evaluation results underscore the efficacy of the improved system, highlighting its superior accuracy and the ability in answering complex questions compared to its predecessor.

## Introduction

With the rapid progression of digital technologies, the chemistry sector is generating vast amounts of complex data. Traditional methods for information storage and retrieval are struggling to manage this increasing volume and complexity. The fragmented and often incomplete state of chemical data also poses significant challenges for machine learning. While machine learning has the potential to revolutionize data analysis, its application is inhibited by its need for large quantities of clean data. Much time is often wasted by simply gathering and cleaning data. This accentuates the importance of accurate and well-structured data.^1,2^

In this regard, since the landmark publication by Berners-Lee et al.,^3^ semantic web technologies and knowledge graphs (KGs) have been introduced as a solution, providing an effective framework for semantic information retrieval. A KG is a network of data expressed as a directed graph, where the nodes of the graph are concepts or their instances (data items), and the edges of the graph are links between related concepts or instances. KGs are often built by using the principles of linked data. KGs enable efficient storage and retrieval of interconnected web-scale data and can identify new relationships among various entities. While they may not necessarily speed up queries or reduce hardware requirements, they have the ability to streamline data discovery and cleaning processes, leading to more efficient data utilization and processing. Major existing KGs include the Wikidata KG,^4^ the DBpedia KG,^5^ and the Google KG.^6^ In the chemistry field, where the need of clean and well-structured data is of fundamental importance, KGs have recently begun to be explored in the context of drug and material discovery and have the potential to assist in key challenges such as target identification.^7,8^

However, querying a KG can be often challenging due to its size and complexity. To submit a query, the user needs to know the formal query language SPARQL^9^ and a complete understanding of the KG schema. In some cases of user error, the queries return no data but are considered formally correct and no warnings are reported.

For this reason, a more user-friendly interface that can retrieve data from KGs is desired. Knowledge graph question answering (KGQA) allows one to answer natural language queries posed over the KG. In particular, KGQA in the chemistry domain is a promising area of research owing to the rapid growth of chemistry-related KGs and the potential advantages of a deep search of the chemical space. By providing a more intuitive interface for querying complex data sets, these technologies have the potential to revolutionize how researchers across various fields interact with and extract value from data.

Marie^10−12^ is a KGQA system for chemistry. It is part of a wider effort to develop a user-friendly interface to interact with The World Avatar (TWA)—a world model built upon a dynamic KG with the aim to interoperate across heterogeneous ontologies and disparate domains, including not just chemistry but also power systems, 3D models of cities and landscapes, among others.^13^ Interactions with TWA are facilitated by agents, which are applications and services capable of performing various operations upon the underlying KG. Navigating the suite of agents for varying use cases can be challenging. Ultimately, a natural language interface for TWA could offer a single unified entry point for user interactions by providing a layer of abstraction on top of the many services and data stores available.

The first iteration of Marie^10^ was an early attempt at developing a multiontology KGQA system for chemistry and demonstrates the viability of converting questions in natural language to SPARQL queries by matching user’s intention to a predefined query template and populate the template with values extracted from user’s utterance. This template-based approach^14−16^ is a traditional method falling under the category of semantic parsing, which aims to parse a given question into its logical form, *i.e.*, a semantically equivalent representation either in SPARQL or an intermediate form that can be trivially converted to SPARQL.^17,18^ The said system’s reliance on hand-crafted templates, which are limited to simple one-hop questions, hampers scalability because the task of expanding the template store falls on domain experts.

Doing away with the need for explicit logical forms, information retrieval approaches rely on signals obtained from input questions to enumerate candidate answers, rank them, and return the top candidate. Inspired by growing research in information retrieval-based QA systems^19−23^ and transfer learning methods using pretrained language models (PLMs), the Marie and BERT system^12^ leverages the PLM BERT^24^ and KG embedding techniques to map KG constituents and natural language questions into low-dimensional vector spaces where ranking of candidate answers can be efficiently performed as vector operations. However, KG embeddings are generally expensive to train and have to be retrained whenever new factual assertions are added to the knowledge store. Although there is a line of research that studies KG reasoning with unseen entities to address these challenges through generalization,^25,26^ it has yet gained adoption by the KGQA community. We hypothesize that this is due to such foundational KG models focusing predominantly on structural information, neglecting the semantic content vital for aligning natural language with KGs. This gap highlights the necessity for further exploration into how foundational KG models can be effectively applied in KGQA, potentially through fine-tuning biencoder architecture that integrates pretrained text and KG encoders or developing cross-encoder architecture for joint processing. Additionally, it is not a trivial task to find a KG embedding model suitable for a given ontology. In fact, a poor modeling strategy can inhibit not only the end performance of KGQA systems but also the extent of complexity that reasoning can be done in the embedding space; as a case in point, the Marie and BERT system^12^ is unable to handle questions with more than one relation. Additionally, as noted by Yu et al.,^27^ information retrieval-based systems tend to be outperformed by their semantic parsing-based counterparts.

More is left to be explored with semantic parsing-based approaches and the use of PLMs. Key approaches to KGQA that have benefited from generative PLMs include end-to-end translation,^28−30^ the retriever-reader model,^31−35^ and dynamic logical form induction.^36,37^ Even though many of these systems have clinched state-of-the-art results on public benchmarks,^30,34,37^ attempts to quantify their speed to demonstrate practical usage are still inadequate; we are only able to find reports of system speed measured on GPU in two papers.^36,37^ This casts doubt on whether systems with impressive accuracy can be realistically deployed in a real-world setting, where specialized hardware might not be available. More broadly, we notice that efforts to study the accuracy-latency trade-off for KGQA in a hardware-constrained setting are lacking despite the wealth of similar work in other machine learning domains.^38−42^

The exploration of data retrieval methods in the context of KGQA introduces a critical comparison between querying KGs and utilizing retrieval-augmented generation (RAG) agents like PaperQA.^43^ KGs are lauded for their precision and structured knowledge, serving as an ideal solution for tasks that demand specific facts or relationships. Their efficient and interpretable framework facilitates direct access to curated information constrained by the scope of their content. Regular updates are imperative to incorporate new knowledge, posing a challenge to maintaining their comprehensiveness. Conversely, RAG agents harness the vast expanse of data within large data sets or corpora, enriching their generative models to produce contextually rich and coherent responses. This flexibility makes RAG agents particularly suited for tasks that benefit from narrative content or require integration of the latest information. However, the efficacy of RAG agents is directly tied to the volume and quality of data that they can access, a process that is both resource-intensive and time-consuming. The reliance on large language models (LLMs) for generation often necessitates the use of GPUs for inference within acceptable latency thresholds or the engagement of external services. Furthermore, the iterative refinement of the data quality and relevance for generation augmentation through LLMs introduces additional latency and hardware demands. In disciplines such as chemistry, where accuracy and specificity are paramount and the need for narrative is less pronounced, KGs emerge as the preferable choice. Their ability to provide precise, structured information aligns well with the field’s requirements, offering a streamlined and efficient method for data retrieval and analysis.

The purpose of this paper is to present a new version of Marie, a KGQA system that aims to empower researchers with varying levels of expertise to explore and utilize chemical data effectively, making it relevant not only to specialists in chemical informatics but also to the wider scientific community. The proposed design of Marie aims to translate questions in natural language to SPARQL queries with the use of a fine-tuned Flan-T5 model,^44^ known for its demonstrated versatility and efficacy in adapting to downstream tasks^44^ while capable of running inference on consumer-grade hardware. Our system adopts a unified pipeline for KGQA, wherein translation is performed end-to-end and without separate components for entity and relation linking but with simple corrective procedures to counteract the uncertainty in the generation of target queries. We also explore 8-bit quantization as a technique to negotiate the trade-off between accuracy and latency. The developed KGQA system is lightweight and able to not just handle multihop questions and but also balance between correctness and speed in a CPU-only setting. This new approach offers significant advantages over the prior implementation that relies on KG embeddings.^12^ Specifically, the updated system boasts higher accuracy and greater flexibility in accommodating changes and evolution of the data stored in the KG without necessitating retraining. Moreover, our system offers a lightweight solution to querying chemical data by leveraging KGs as the structured data store crafted with supervision by human experts, whereas RAG agents generally relegate to LLMs the task of data analysis and information extraction, which demand specialized hardware or external services to render real-world usage viable.

## Related Work

### PLMs for Knowledge Graph QA

In this section, we provide an overview of major approaches that leverage PLMs for KGQA and where our system stands in this suite of methods.

#### End-to-End Translation

Capitalizing on the generative capabilities of LMs, KGQA systems that adopt the translation approach take in natural language questions and feed them into fine-tuned LMs to output SPARQL queries. For many systems,^29,33^ the grounded queries are generated in a one-shot manner. Meanwhile, LMs could be employed to obtain only query skeletons, which are then grounded with entities and/or relations detected by a separate set of components.^28,45^ Recognizing that one-shot generation of executable SPARQL queries is prone to KG misalignment especially for unseen entities and relations, but decoupling entity and relation linking from logical form generation opens up more room for errors, researchers behind the system GETT-QA^30^ propose a middle ground, whereby in the first step, SPARQL queries are generated with entity and relation slots already filled with their surface forms as found in input questions, and in the second steps, these labels are grounded to actual KG entities and relations.

#### Retriever-Reader Model

Similar to text-to-text translation, systems that follow the retriever-reader model also utilize fine-tuned LMs to generate SPARQL queries but augment the input into the LMs with additional signals. The pipeline of such systems can be broken down into two main steps: in the first step, a retriever processes an input question to gather information relevant to the formation of the corresponding logical form; in the second step, a reader, which is often a fine-tuned LM, takes in the given question and the retrieved information and outputs the desired logical form. Different systems design for different kinds of information to be retrieved and fed into the reader, such as entities and relations detected from input questions,^31,34,46^ candidate logical forms,^32^ candidate query paths,^34,35^ or linearized facts.^27^ To ensure that the generated queries are compliant with the ontology of the KG, many of these systems impose decoding constraints on the reader^34^ or perform an additional step of revision to realign output logical forms to the KG’s ontology.^31,32^

#### Dynamic Logical Form Induction

Rather than directly generating formal queries in full, systems that perform dynamic logical forms induction starts with a partial query and incrementally expands it until the desired logical form is found using the discriminative ability LMs to guide this construction process.^36,37^ The incremental expansion of logical forms enables more fine-grained control over the generation process by limiting the search space of candidate query paths and enforcing grammatical rules.

#### Relevance to Our System

It is critical to assess the applicability of the aforementioned approaches with respect to the development of a KGQA system for chemistry. Although dynamic logical form induction has yielded state-of-the-arts results,^37^ this approach relies strongly on the completeness of the KG to construct logical forms. For instance, for a rare chemical species, it is not uncommon that data about some of its properties are unavailable. In such a scenario, the dynamic logical form induction approach would fail to generate the appropriate logical form and potentially report its failed prediction, while in fact, the expected behavior would be to produce a valid SPARQL query and return an empty response. End-to-end translation and retriever-reader approaches are both promising, differing only in whether the input into LMs is augmented with extra information other than the question posed to the system. In this work, we adopt the end-to-end translation approach and draw inspiration primarily from GETT-QA^30^ due to its straightforward design and leave the exploration of retriever-reader model in future work.

### TWA Chemistry Ontologies

Marie is a KGQA system developed for chemistry, which operates on top of the TWA KG chemistry domain. The TWA KG is a comprehensive, cross-domain, and dynamic KG adhering to linked data principles. It seamlessly integrates various ontologies, such as OntoSpecies,^47^ OntoKin,^48^ OntoCompChem,^49^ OntoPESScan,^50^ and OntoMOPs,^7^ all tailored for representing chemical information. OntoSpecies is a fundamental chemistry ontology within TWA KG. This ontology contains the IRIs of about 36,000 chemical species and is constantly updated with new entries. The ontology also covers the basic chemical and physical properties of the species. It encompasses a diverse collection of identifiers, classifications, and uses of chemical species, as well as spectral data, in addition to information indicating its origins and attribution. OntoKin is an ontology focused on representing chemical kinetic reaction mechanisms, offering details on reactants and products as well as kinetic, thermodynamic, and transport models.^48^ OntoCompChem is an ontology designed for computational chemistry calculations, particularly for density functional theory calculations.^49^ OntoCompChem currently represents single-point calculations, geometry optimizations, and frequency calculations. A different ontology, OntoPESScan, has been specifically designed for the representation of potential energy surface (PES) scans.^50^ OntoMOPs is an ontology designed for the rational design of metal–organic polyhedra (MOPs).^7^ It encodes assembly models and generic building units as blueprints for creating MOPs, facilitating their design with chemical and spatial reasoning. The current iteration of Marie is specifically configured to operate within the OntoSpecies KG. In this research paper, we aim to showcase the utility of a fine-tuned language model for translating natural language queries into SPARQL queries, particularly for nonshallow ontologies. This approach enables us to effectively address complex questions within the domain effectively. However, our ultimate goal is to broaden the scope of Marie in future iterations to encompass the entire TWA chemistry domain.

### Methodology

Our system comprises three main steps: preprocessing, translation, and postprocessing. In the preprocessing step, special characters present in the input question are encoded and physical quantities are converted to SI units. In the translation step, the preprocessed question is passed to a fine-tuned Flan-T5 model to generate the SPARQL query. In the last step, the predicted query is postprocessed to have special characters decoded and additional triple patterns added to enhance user experience. We describe our system architecture in greater detail in Section “System Architecture”. Additionally, to train a translation model that learns a mapping from natural language to formal queries, a supervised data set of question-logical form pairs is needed. Our procedure for constructing this data set is outlined in Section “Dataset”.

### Data Set

**Figure 1:**
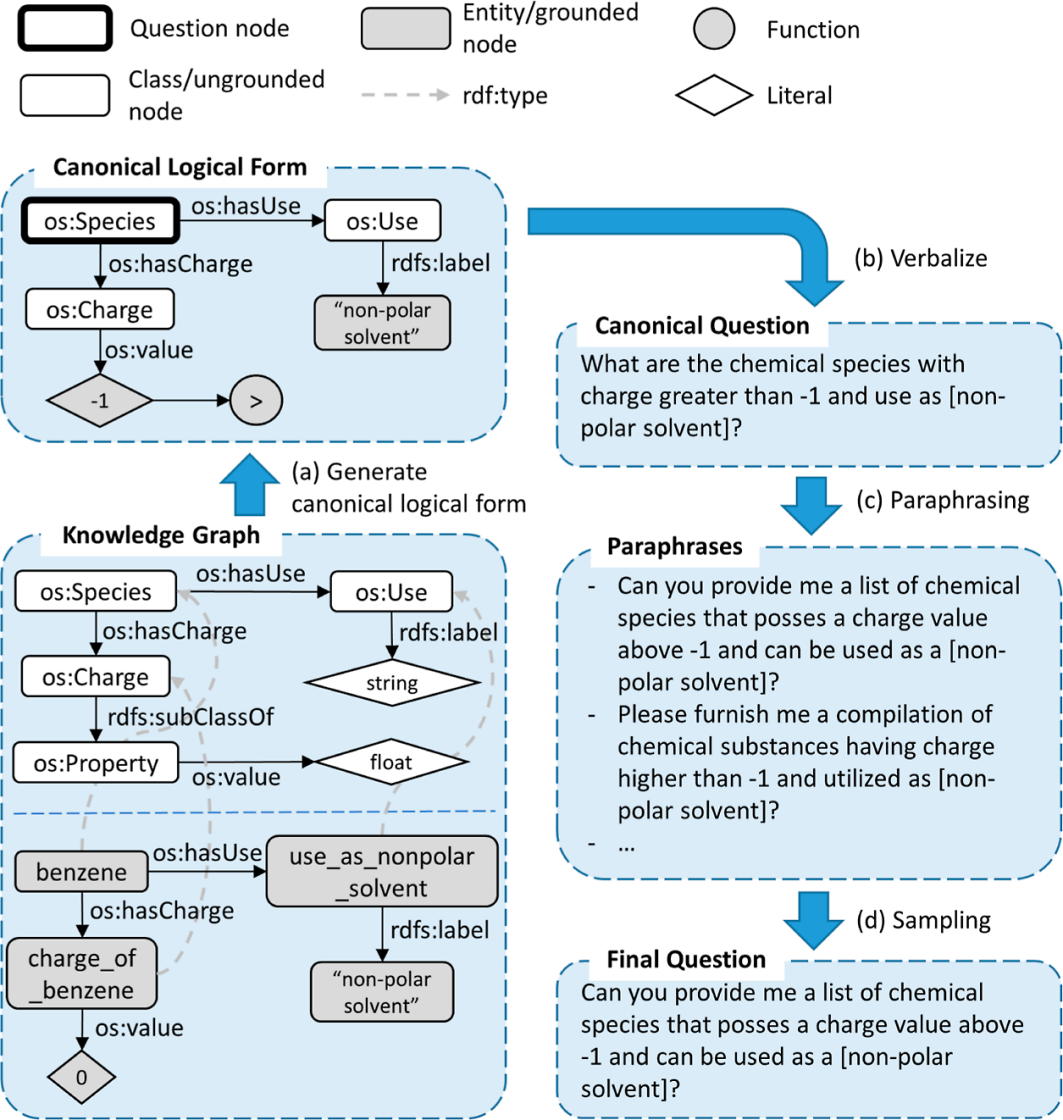
Steps to generating our data set. (a) A canonical logical form is generated from a schema subgraph and its instantiation. (b) The logical form is verbalized to form a canonical question. (c) The canonical question is rephrased into different utterances. (d) The final question is sampled from the pool comprising the canonical question and its valid paraphrases.

The scope of our data set includes information from OntoSpecies KG about chemical species such as their chemical and physical properties, applications, and chemical classifications. Following landmark works in constructing data sets for KGQA,^18,51^ we devise a data construction pipeline that runs in three main steps: (1) generate logical forms from a KG, (2) verbalize the logical forms into questions in natural language, and last, (3) rephrase the verbalized questions to obtain examples with more linguistic variability. Figure 1 illustrates the data generation process in a nutshell.

### Canonical Logical Form Generation

**Figure 2:**
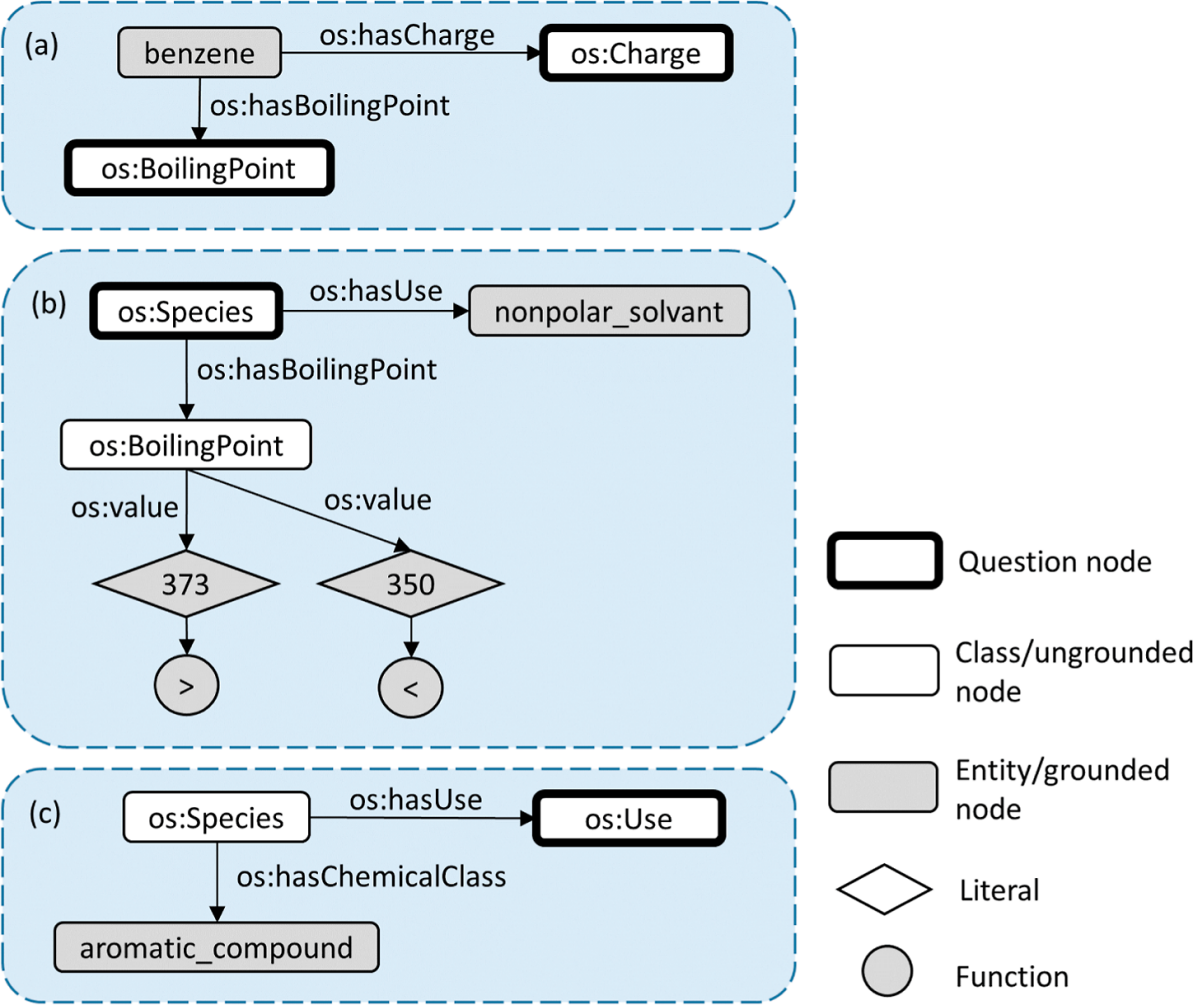
An overview of query types included in our data set. (a) One-hop queries that look up information about a given chemical species or more, *e.g.*, “what are the boiling point and charge of benzene?”. (b) One-hop queries that find chemical species based on specified criteria, *e.g.*, “what are the chemical species that can be used as a nonpolar solvent and have a boiling point between 373 and 350?”. (c) Two-hop queries that ask about properties of chemical species belonging to a particular chemical class, *e.g.*, “what are the applications of chemical species classified as aromatic compounds?”.

Emulating the methods used in the creation of general-domain KGQA data sets GrailQA^52^ and GraphQuestions,^18^ we traverse the KG’s ontology to obtain ungrounded subgraphs consisting of classes and their relations; these subgraphs subsequently have some of their nodes grounded and functions added, yielding canonical logical forms. We control the level of complexity of our generated queries by applying some heuristics to the structure of the retrieved subgraphs and the choice of grounded and question nodes. The out-degree of the os:Species is chosen to vary from 1 to 3, and the grounded and question nodes are chosen such that we obtain three types of queries: those that retrieve data about a specified chemical species or more, those that search for chemical species that satisfy a set of conditions constrained upon their properties, and those that inquire about the properties of chemical species belonging to a given chemical class. See Figure 2 which provides examples of these query types.

In contrast to the aforementioned general-domain KGQA data sets that include up to only one function that can be a counting operation, a superlative (arg max, arg min), or a comparative (>*rbin*, ≥, <, ≤),^18,52^ we apply one comparative operator out of the five choices shown in Table 1 on all numerical qualifiers. The rationale for this is to accommodate practical scenarios of data lookup in the chemistry domain, such as the use case of finding solvents whose boiling point falls within a certain range when performing distillation.

**Table 1:** Comparative Operators Present in Our Data Set

	logical form	verbalization
higher	*x* > *a*	higher than *a*
lower	*x* < *a*	lower than *a*
inside	*a* < *x* < *b*	inside the range between *a* and *b*
outside	*x* < *a*∥*x* > *b*	outside the range between *a* and *b*
around	0.9a < *x* < 1.1a for a > 0	around *a*

Unlike most KGQA data sets, we do not include entity IRIs in our generated SPARQL queries and instead perform entity linking directly within the SPARQL queries by exact string matching with node labels; for the case of entities of the class os:Species, linking is done by exact matching with any of its chemical identifiers of any subclass of os:Identifier.

### Verbalization

To support the different ways that a query intent can be formulated and submitted to QA systems, we consider three kinds of verbalizations: the interrogative form, the imperative form, and the keyword search. Each of these yields a different canonical question; see Table 2 for an example.

**Table 2:** Overview of Verbalization Types Used for the Formulation of Canonical Questions

verbalization type	example
interrogative form	“what is the charge of benzene?”
imperative form	“tell me about the charge of benzene.”
keyword search	“charge of benzene”

### Paraphrasing

Paraphrasing serves the purpose of capturing different sentence structures that express the same query intent as well as the various surface forms that an entity or relation can appear in. For example, the two questions “what is the boiling point of water?” and “at what temperature does water boil?” correspond to the same SPARQL query involving a single hop over the relation os:hasBoilingPoint.

With the assumption that entity linking is done by exact string matching of detected entity spans, we do not alter entity mentions in query verbalizations during paraphrasing; in other words, only the surface forms of relations are rephrased. We perform paraphrasing to only verbalizations in the interrogative and imperative forms using OpenAI’s chat completion API with the gpt-3.5-turbo model. For each canonical question, five paraphrases are generated, which are manually checked for semantic correctness; paraphrases that deviate from the original meaning are rejected. Last, the final question is sampled from a pool comprising the canonical question and its valid paraphrases.

### Data Set Analysis

Three data sets are generated: (1) the train set, which is used for fine-tuning of PLMs; (2) the dev set, which helps with model selection during the fine-tuning process; and (3) the test set, which enables unbiased evaluation of fine-tuned models. In total, our data set covers 56 relations defined in the ontology of OntoSpecies. See Table 3 for the statistics on some characteristics of our data set.

**Table 3:** Statistics on Some Characteristics of Our Data Set

		**% of examples with***x***relations**			% of function occurrence				
	size	*x* = 1	2	3	higher	lower	inside	outside	around
train	1608	57.84	37.25	4.91	8.27	8.77	7.59	8.58	9.08
dev	180	65.56	32.22	2.22	11.11	6.11	8.33	8.89	7.78
test	182	63.73	33.52	2.75	10.44	9.34	7.69	8.79	7.14

### System Architecture

Flan-T5,^44^ which is the improved version of its predecessor T5,^53^ is our model of choice. The T5 model family has been mobilized in several KGQA systems^27,30,31,34,35,46,54^ owing to its open-source nature and the ability to run on consumer-grade hardware.

**Figure 3:**
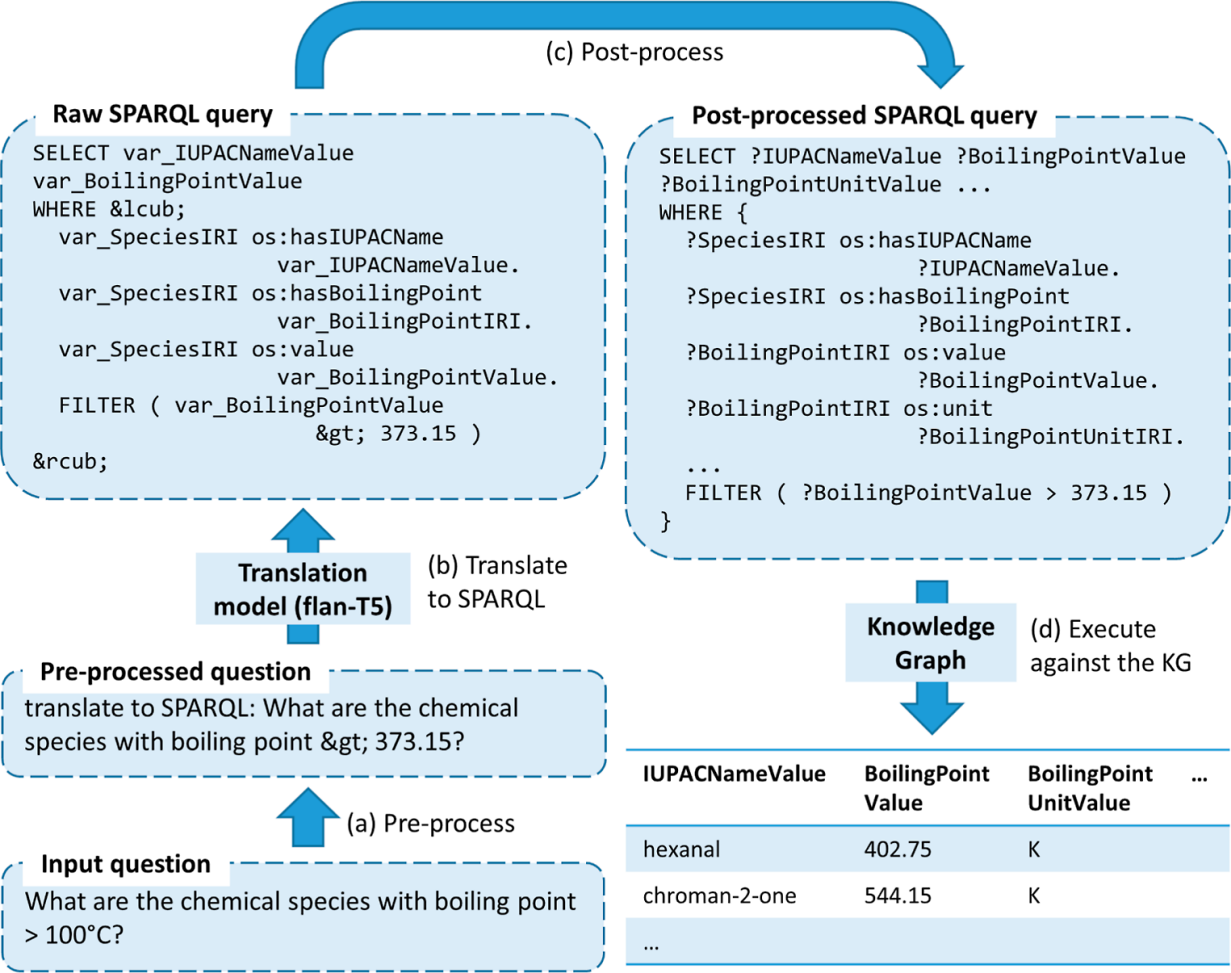
Key steps in our KGQA system. (a) Physical quantities in the input question are converted to SI units, special characters are encoded, and an instruction prompt is prepended. (b) The question is fed into a fine-tuned Flan-T5 model to obtain SPARQL translation. (c) Special tokens in the translated query are decoded, and additional node patterns are added to enhance user experience. (d) The query is executed against the KG to obtain the results.

One issue with T5 is that it is trained specifically on natural language texts, thus the T5 tokenizer treats characters such as the less-than sign (“<”) and the curly braces (“{” and “}”) as unknown tokens. To handle this out-of-vocabulary problem, we map these characters into alternative string representations, specifically their corresponding HTML entities (“lt;”, “lcub;”, “rcub;”). For symmetry, we also convert the greater-than sign to its HTML entity counterpart (“>” is substituted with “gt;”). Below are outlined the additional preprocessing and postprocessing operations employed by our system. See Figure 3 for a running example of a query passing through the KGQA system.

### Preprocessing of Input Texts

Physical quantities are meaningful only as long as their units are specified, and different users under varying scenarios might have their own preferred unit system that they find more convenient to work with. To facilitate interoperability with different unit systems, we convert any mentions of physical quantities in the input questions to SI units, which are the unit system used by the OntoSpecies KG. Here, we assume that units indicated by the user are always valid for the invoked physical quantities, e.g., when talking about temperatures, the user would use degree Celsius, degree Fahrenheit, or Kelvin and not kilogram.

Following unit conversion, characters that are out of T5′s vocabulary are encoded into HTML entities as aforementioned. Last, to keep in line with the training and fine-tuning approaches of Flan-T5,^44,53^ we prepend the input question with the instruction prompt “translate to SPARQL: ”.

### SPARQL Encoding

SPARQL encoding determines the representation of SPARQL queries that the language model learns. Besides encoding out-of-vocabulary characters, we follow^55^ in converting the prefix marker of query variables, which is the question mark character ?, to the string prefix var_. As mentioned earlier, the design of T5 training and tokenization and its intended application is for natural language. With the assumption that the syntactical function of a question mark is to delimit the end of an interrogative statement, the T5 tokenizer by default strips all whitespaces that appear before a question mark. When this behavior is applied to SPARQL, a triple pattern such as “a ?b c” is read by the model as “a?b c”, rendering the role of the question mark ambiguous: is it a prefix, a suffix, or a delimiter? To clearly designate the SPARQL syntax for query variables, we instead use a string prefix. See Table 4 which provides an illustration.

**Table 4:** Example of How a SPARQL Query Is Encoded in Our KGQA System^a^

original SPARQL query
SELECT ?SpeciesIRI ?BoilingPointValue
WHERE {
?SpeciesIRI os:hasBoilingPoint ?BoilingPointIRI
?BoilingPointIRI os:value ?BoilingPointValue
FILTER (?BoilingPointValue > 373)
}

encoded SPARQL query
SELECT **var_**SpeciesIRI **var_**BoilingPointValue
WHERE **&lcub;**
**var_**SpeciesIRI os:hasCharge **var_**BoilingPointIRI
**var_**BoilingPointIRI os:value **var_**BoilingPointValue
FILTER (**var_**BoilingPointValue > 373)
**&rcub;**

a The substitutions are in bold.

### Postprocessing of Predicted SPARQL Queries

SPARQL queries generated by the language model are in the encoded representation, as specified in “SPARQL encoding”. Therefore, they are first decoded to recover the original form. The subsequent postprocessing procedures are as follows.

#### Triple-Pattern Expansion

In theory, the retrieval of entity nodes as answers is sufficient for QA. However, users might require additional information for the answers to be meaningful and human-readable. For instance, when inquiring about the boiling point of a chemical species, it is important to also display the reference state at which the boiling point is measured. Therefore, after decoding SPARQL queries predicted by our translation model, we augment them with additional triple patterns, depending on the class of the answer nodes. See Table 5 for an example.

**Table 5:** Example of Triple-Pattern Expansion in Our Postprocessing Step^a^

SPARQL query with minimal triple patterns
SELECT ?SpeciesIRI ?BoilingPointValue
WHERE {
?SpeciesIRI os:hasBoilingPoint ?BoilingPointIRI
?BoilingPointIRI os:value ?BoilingPointValue
FILTER (?BoilingPointValue > 373)
}

SPARQL query with expanded triple patterns
SELECT ?SpeciesIRI **?IUPACNameValue** ?BoilingPointValue
**?UnitValue ?RefStateValue ?RefStateUnitValue**
WHERE {
**?SpeciesIRI os:hasIUPACName ?IUPACNameIRI**
**?IUPACNameIRI os:value ?IUPACNameValue**
?SpeciesIRI os:hasBoilingPoint ?BoilingPointIRI
?BoilingPointIRI os:value ?BoilingPointValue
**os:unit ?UnitIRI**
**?UnitIRI rdfs:label ?UnitValue**
**OPTIONAL {**
**?BoilingPointIRI os:hasReferenceState ?RefStateIRI**
**?RefStateIRI os:value ?RefStateValue**
**os:unit ?RefStateUnitIRI**
**?RefStateUnitIRI rdfs:label ?RefStateUnitValue**
}
FILTER (?BoilingPointValue > 373)
}

a The added query variables and triple patterns are in bold.

#### Copy Correction

Our preliminary experiments show that PLMs face difficulties copying exact text spans of chemical species when the surface forms are long, repetitive patterns, as illustrated by Table 6. While researchers have come up with elaborate copy mechanisms that often involve reworking network architecture,^33,56−58^ we make no alteration to the underlying translation model. Instead, we assume that the model can detect correct text spans but might not be able to generate them with absolute copying fidelity. To rectify the copy error, we match the model’s generated text spans with the closest substrings of the input question using the Levenshtein distance as the distance metric.

**Table 6:** Example of a Question about a Chemical Species Represented by Its SMILES String^a^

Question	share information regarding the optical rotation of **CC1C(C(CC(O1)OC2C(OC(CC2O)OC3C(OC(CC3O) OC4CCC5(C(C4)CCC6C5CCC7(C6(CCC7C8=C**C(=O)**OC8)O)C)C)C)C)O)O**
SPARQL query	SELECT ?OpticalRotationValue WHERE {VALUES (?species) { (“**CC1C(C(CC(O1)OC2C(OC(CC2O)OC3C(OC(CC3O) OC4CCC5(C(C4)CCC6C5CCC7(C6(CCC7C8=C**C(=O)**OC8)O)C)C)C)C)O)O**”) } ?SpeciesIRI ?hasIdentifier ?IdentifierIRI. ?IdentifierIRI os:value ?species. ... }

a The corresponding SPARQL query has to copy the string span exactly.

#### Relation Correction

We follow^30^ in realigning predicted relations to the actual ones in the ontology of OntoSpecies using an embedding matching mechanism. We employ the Sentence-BERT^59^ model, which has been trained using a Siamese network for the semantic matching task, to map relations to low-dimensional vector representations and match them using the cosine similarity as the distance measure.

### Fine-Tuning

For fine-tuning, we update all model parameters. We use the AdamW optimizer^60^ with a learning rate of 2 × 10^–4^ and ε = 1 × 10^–6^. We kept a constant batch size of 32 across experiment runs and adjusted the number of gradient accumulation steps as needed. We train for a fixed number of three epochs and perform no hyper-parameter tuning. All fine-tuning is done in a distributed, data-parallel setting under a hardware budget of a single node consisting of 4× NVIDIA A100-SXM-80GB GPUs.

### Evaluation

#### Translation Quality

The translation quality of our system is evaluated using two automated metrics: the SacreBLEU score^61^ and accuracy. SacreBLEU is a variant of the popular BLEU score used for the evaluation of machine translation systems by comparing token-level similarity between reference texts and candidate texts; SacreBLEU has been preferred over BLEU for the former’s reproducibility and ease of comparison. The metric runs on a scale from 0 to 100, with higher values indicating higher degrees of lexical match. For translation accuracy, we assign a score of 1 if the machine-translated output is an exact word-level match with the gold reference.

#### Quantization

Quantization is the technique that maps floating point values to a quantization space supported by fewer bits. This is an established method to significantly reduce inference latency and memory requirement of neural networks with manageable impacts on accuracy.^62−67^ We employ 8-bit dynamic quantization, whereby the weights of neural networks are quantized to 8-bit integers before test time, and activations are dynamically quantized during inference. We evaluated the performance of our system under two settings of floating point precision: full precision (32-bit) and 8-bit quantization.

#### Hardware

Fine-tuned models are evaluated in a CPU-only setting with a 12th Gen Intel(R) Core(TM) i7-1270P 2.20 GHz processor and 64GB RAM. To mimic a production environment, inference is run with a more performant model format and interpreter, which are chosen to be the ONNX format^1^ and the ONNX Runtime^2^.

## Results and Discussion

### Quantitative Results and Error Analysis

Table 7 shows the quantitative results for the translation quality of our KGQA system with different base models and postprocessing procedures. These results indicate that larger models are able to attain higher accuracies and benefit less from manually crafted postprocessing steps. The fine-tuned Flan-T5-XXL model, which has three billion parameters, attains a near-perfect translation accuracy score of 98.90%.

**Table 7:** Translation Qualities for Various Fine-Tuned Models^a^

model	SacreBLEU	Δ	accuracy (%)	Δ
Flan-T5-small (60M)	78.13		36.26	
+ copy correction	77.86	–0.27	37.36	+1.10
+ relation correction	79.79	+1.93	43.96	+6.60
Flan-T5-base (250M)	95.43		63.19	
+ copy correction	95.46	+0.03	63.74	+0.55
+ relation correction	96.12	+0.66	68.13	+4.39
Flan-T5-large (780M)	98.75		92.31	
+ copy correction	98.75	+0.00	92.31	+0.00
+ relation correction	99.21	+0.46	95.60	+3.29
Flan-T5-XL (3B)	99.56		95.60	
+ copy correction	99.56	+0.00	97.25	+1.65
+ relation correction	**99.69**	+0.13	**98.90**	+1.65

a The number of parameters of base models are given in brackets next to their names.

To better understand the capacity of PLMs to learn mappings between natural language questions and SPARQL queries in the chemistry domain and the effect of scaling the model size, we conduct an error analysis that classifies incorrect predictions by aspects of logical forms, as summarized in Table 8. We observe that while a small model such as the Flan-T5-small variant, which has only 60 million parameters, is able to learn the surface representations of SPARQL to some extents, as seen in the unadjusted SacreBLEU score of 78.13, it fails to acquire the implicit syntactical rules of SPARQL, resulting in 10.99% of predictions with incorrect SPARQL syntax; meanwhile, this figure drops to zero for the Flan-T5-XL variant. Smaller models are also less able to handle the diverse query structures that our data set encompasses and perform poorer at relation prediction. Of note is that smaller models such as Flan-T5-small and Flan-T5-base face difficulties learning the mappings for comparative operators, especially when disambiguating the “inside” and “outside” functions.

**Table 8:** Percentages of Incorrect Predictions Classified by Aspects of Logical Forms

	% of incorrect predictions			
model	**syntax**	**query structure**	**relation**	**function**
Flan-T5-small	10.99	24.18	7.69	17.03
Flan-T5-base	2.75	7.14	4.40	15.93
Flan-T5-large	1.10	1.65	2.20	0
Flan-T5-XL	0	0.55	0.55	0

### Accuracy-Latency Trade-Off

Next, we quantify the translation latency for varying model sizes and quantization settings. As expected, larger models, although better at natural language-to-SPARQL translation, require more resources to run inference. As can be seen in Table 9, while larger networks produce more accurate results—about 25% increment in accuracy for every increase in the scale of the base model—their translation latency is slower by 2.5–3 times, and their memory consumption rises by 1.5–2.5 times.

**Table 9:** Trade-Offs between Translation Accuracy, Translation Latency, and Memory Consumption with Varying Neural Network Sizes and Quantization Treatment, in a CPU-Only Setting^a^

model	accuracy (%)	Δ	translation latency (s)	speedup	memory (GiB)	%Δ
Flan-T5-small	43.96		1.15		3.18	
+ quantization	21.98	–21.98	0.79	×1.46	2.76	–13.21
Flan-T5-base	68.13		3.00		4.32	
+ quantization	66.48	–1.65	1.72	×1.74	3.29	–23.84
Flan-T5-large	95.60		8.24		7.70	
+ quantization	96.70	+1.10	4.05	×2.03	4.41	–42.73
Flan-T5-XL	98.90		25.15		19.21	
+ quantization	89.91	–10.99	9.48	×2.65	8.55	–55.49

a As translation latency varies with query complexity, see Figure S1 for its distribution.

Experiments with quantized models at test time reveal that quantization can significantly reduce the inference time and memory requirement while maintaining similar levels of accuracy for medium-sized neural networks. Particularly, quantized Flan-T5-base and Flan-T5-large models experience slight deviations to their accuracy within a 2% margin, yet their translation latency is sped up by up to 2 times and their memory consumption drops by up to 40%. Furthermore, the narrow increase in accuracy observed for the Flan-T5-large variant indicates that quantization could have a regularization effect, thus enabling the quantized model to perform better than the full-precision baseline, similar to previous work in the literature.^68−70^ In contrast, quantification of the Flan-T5-XL variant incurs a 10.99% drop in accuracy. This anomalous deterioration is a recognized phenomenon that emerges in LMs exceeding a certain size.^67^ While there exists quantization schemes to rectify this anomalous behavior,^67^ the ecosystem is still in its nascent stage and the implementation for our specific model architecture and inference runtime is yet developed and rigorously tested. We therefore do not attempt to explore the use of a specialized quantization procedure in our system.

**Figure 4:**
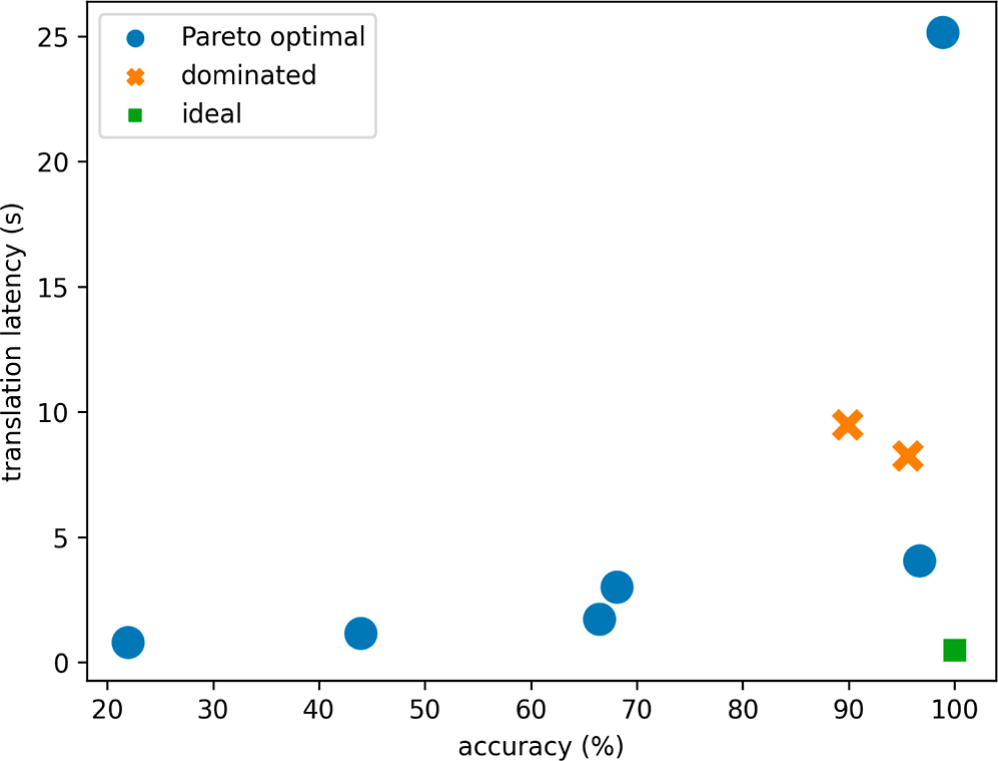
Scatterplot showing the accuracy-latency trade-off of our KGQA system under a CPU-only setting.

Our systematic quantification of accuracy and translation latency helps us establish the Pareto front for the multiobjective optimization of accuracy and latency of our KGQA system, as depicted in Figure 4. This could prove instructive to the practical deployment of a KGQA system. If the Euclidean distance from the ideal point corresponding to 0 latency and 100% accuracy were to be taken as the objective function, *i.e.*, a quadratic objective function, our data support the claim that the quantized Flan-T5-large model provides the optimal compromise between accuracy and speed, striking an amount of CPU memory consumption of less than 5 GiB and an accuracy score of 96.70%.

### Example Use Case and QA System Comparison

OntoSpecies KG was designed to permit complex queries and easy data analysis and processing in the general chemistry domain. The information reported on chemical species can be used to compare chemical properties of similar compounds, find compounds with required characteristics, as well as automate laborious data gathering from research activities.^47^ In this section, we showcase how Marie can be used for some of these tasks without the requirement of SPARQL knowledge and KG schema knowledge.

**Figure 5:**
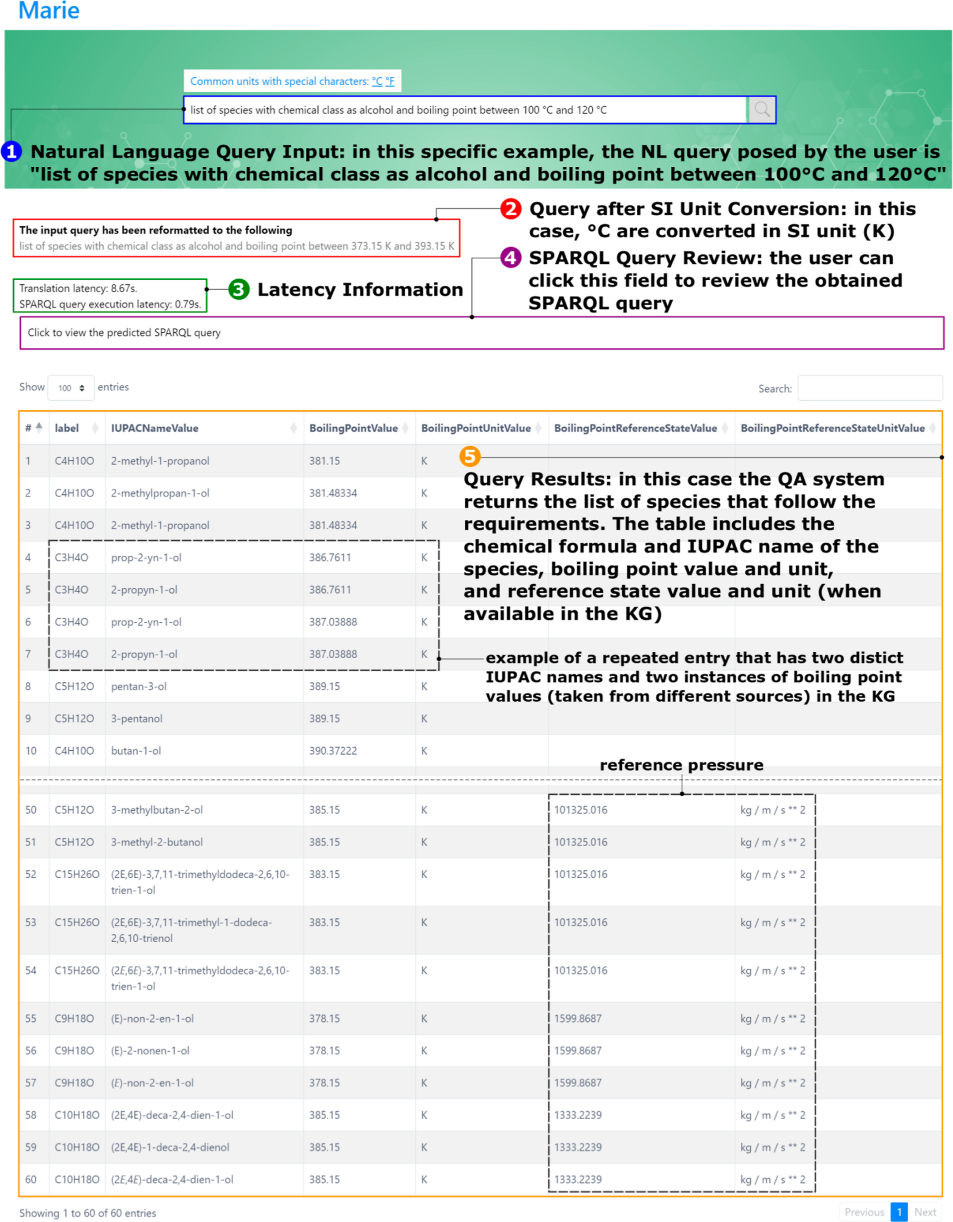
Illustration of Marie’s interface for the query example “list of compounds with chemical class as alcohol and boiling point between 100 and 120 °C”. The figure highlights (1) natural language query input, (2) query after SI unit conversion, (3) latency information, (4) SPARQL query review, and (5) query results.

Take, for instance, the challenge of identifying property trends for specific chemical categories such as the boiling points of species identified as alcohols. Traditionally, this would involve first identifying the species tagged as alcohols and then delving into their associated properties—a process that can be time-consuming when relying on conventional online sources. However, with the integration of KGs and Marie, this task becomes straightforward by simply asking our QA system. An example can be seen in Figure 5 where a screenshot of Marie interface showcases a query made in natural language: “list of compounds with chemical class as alcohol and boiling point between 100 and 120 °C”. Upon entering the question into the query box [as indicated by field (1) in Figure 5], the search engine refines the question to match the SI unit standards. This revised question is then displayed in field (2). The engine subsequently produces a table containing the chemical formula, IUPAC name, boiling point values and units, and, when available in the KG, reference pressure values and units [field (5) in Figure 5]. This outcome is akin to what is obtained from a direct SPARQL query on the KG. Users have the option to review the generated SPARQL query by selecting field (4). Additionally, the time taken for translation and the execution latency of the SPARQL query are displayed in field (3). It is important to mention that each entry in the table is displayed at least twice due to every compound in the KG having two distinct IUPAC names. In some cases, the redundancy extends beyond this because of the manner in which OntoSpecies KG sources its data from PubChem.^47,71^ Given that PubChem aggregates properties from a variety of sources, it is not uncommon to encounter multiple instances of the same property. A case in point is 1-propyn-1-ol, which is listed in Figure 5 with two separate boiling point values, each attributable to a distinct source. As a result, the system fetches every possible combination of the queried data. Nevertheless, in upcoming versions of Marie, we plan to implement a filter in the SPARQL query to prevent the display of duplicate entries.

**Figure 6:**
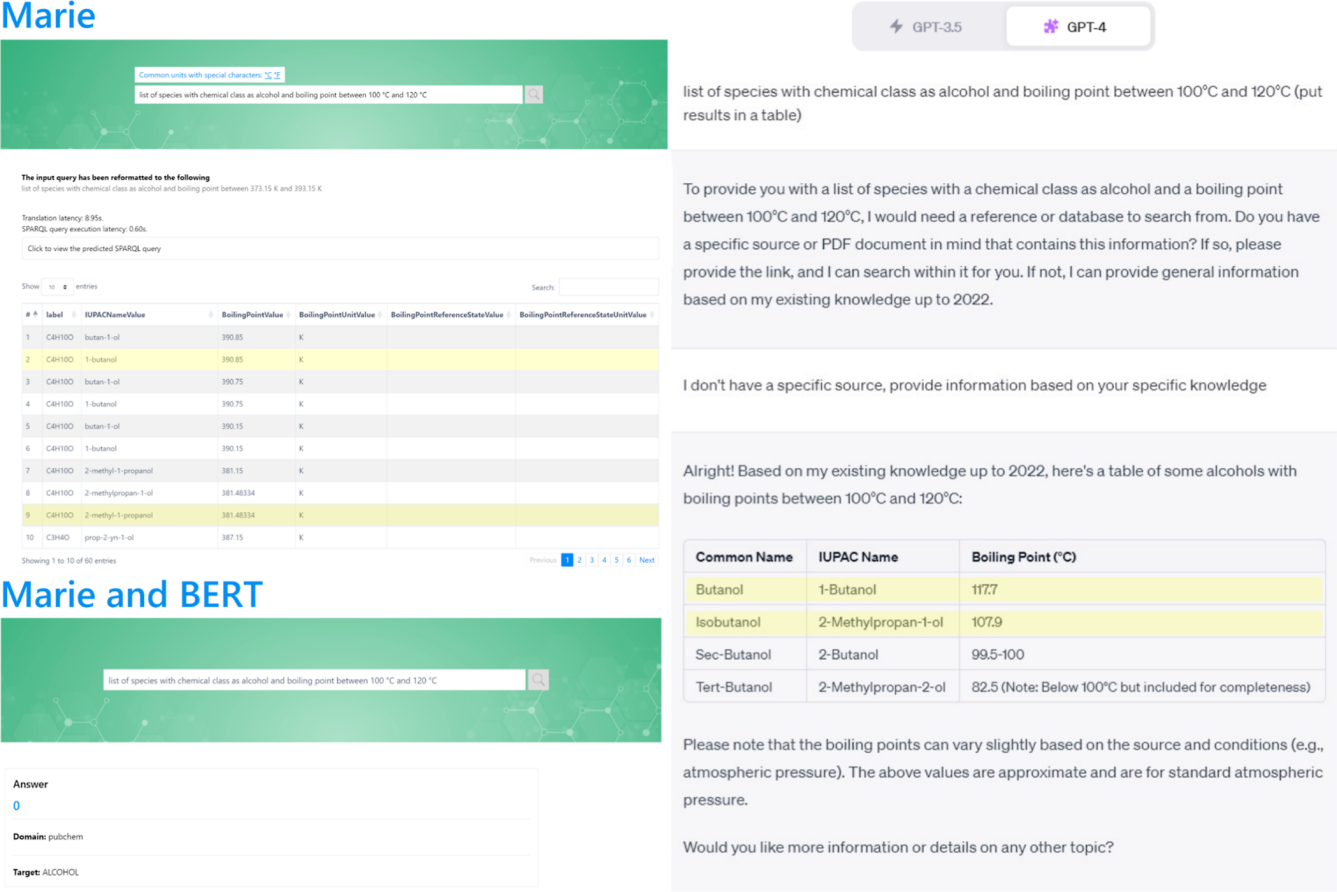
Comparative visualization of responses from Marie (top left), Marie and BERT (bottom left), and ChatGPT-4 (right) to the query “list of compounds with chemical class as alcohol and boiling point between 100 and 120 °C”.

Figure 6 presents a comparative view of responses from Marie, Marie and BERT,^12^ and ChatGPT-4^72^ when posed with the same question. In contrast to our updated version, the earlier Marie and BERT could not provide an answer. This limitation stems from the multihop queries, which our prior version could not handle. Another shortcoming of Marie and BERT is its inability to manage units effectively (questions must be framed using SI units), and it also lacks details regarding the reference state. When a question is posed to ChatGPT-4, the system initially prompts the user to reference a database or upload a specific PDF source. If we indicate that we do not have a particular source and request information based on its inherent knowledge, ChatGPT-4 produces a concise table featuring four species. Notably, two of these species are marginally outside the specified range but are included for the sake of completeness. The remaining two (1-butanol and 2-methylpropan-1-ol) are also returned by Marie (species highlighted in yellow in Figure 6). While ChatGPT-4’s response is more descriptive, correctly uses units, and provides data on the reference state, it is not as comprehensive as one might hope. This highlights the importance of integrating external databases (in our case, OntoSpecies KG) in the QA system to achieve more exhaustive and precise results. Our system excels in delivering precise results and seamlessly adapts to the evolving nature of the database. As the KG expands, the responses generated become increasingly comprehensive and accurate. This dynamic adaptability is a significant advantage as it eliminates the need for periodic retraining. This means that as more data are added or updated in the KG, our system can instantly leverage this new information to provide richer and more informed answers, ensuring that users always have access to the most current and accurate data available.

Users can interact with Marie by following this link (https://theworldavatar.io/chemistry/documentation/marie). However, the system is still under development, and the accuracy of the results will increase with further refinement of the underlying ontologies.

## Conclusions

In this paper, we develop a KGQA system for the chemistry domain that performs end-to-end translation of natural language questions to SPARQL queries with no separate components for entity or relation linking. Additionally, we conduct a hardware-constrained search to find a lightweight configuration for our system that offers a satisfactory compromise between accuracy and speed in a CPU-only setting.

In future work, we will expand the system to work with more ontologies to facilitate a wider range of cross-domain use cases in chemistry-related research and industrial applications.

## Supporting Information Available

Distribution of translation latency with varying base models and quantization settings (PDF)

## Notes

The specific version of the software corresponding to the state at the time of publication can be found in commit “899d404d1b065529397d3d84bbf5acb1a07ecdf2” within the repository.

## Notes

All third-party software used in this system is free and available.

## Footnotes

1 https://onnx.ai/.

2 https://onnxruntime.ai/.
