# Supplementary material for: Leveraging Text-to-Text Pretrained Language Models for Question Answering in Chemistry
Source: ACS Omega. 2024 Mar 12;9(12):13883–96. doi: 10.1021/acsomega.3c08842 (PMC10976360; doi:10.1021/acsomega.3c08842)
Supplement: Supplementary file 1 — ao3c08842_si_001.pdf [file ao3c08842_si_001.pdf]

Supporting Information: Leveraging text-to-text pre-trained language models for question answering in chemistry

Dan Tran,[†] Laura Pascazio,[†] Jethro Akroyd,[†,‡,¶] Sebastian Mosbach,[†,‡,¶] and Markus Kraft*,[†,‡,¶,§,‖]

†*CARES, Cambridge Centre for Advanced Research and Education in Singapore, 1 Create Way, CREATE Tower, #05-05, Singapore, 138602*

‡*Department of Chemical Engineering and Biotechnology, University of Cambridge, Philippa Fawcett Drive, Cambridge, CB3 0AS, United Kingdom*

¶*CMCL Innovations, Sheraton House, Castle Park, Cambridge CB3 0AX, United Kingdom*

§*School of Chemical and Biomedical Engineering, Nanyang Technological University, 62 Nanyang Drive, Singapore, 637459*

‖*The Alan Turing Institute, 96 Euston Rd., London, NW1 2DB, United Kingdom*

E-mail: mk306@cam.ac.uk

Supplementary figures

Due to the cross-attention mechanism for generating next tokens, the runtime of the Flan-T5 decoder grows quadratic with the output sequence. As the SPARQL queries in the test set vary in length depending on their complexity, the decoding time is expected to display considerable deviations from the mean. Therefore, in addition to the mean values indicated in Table 9, we also report the distribution of the translation latency in Figure S1.

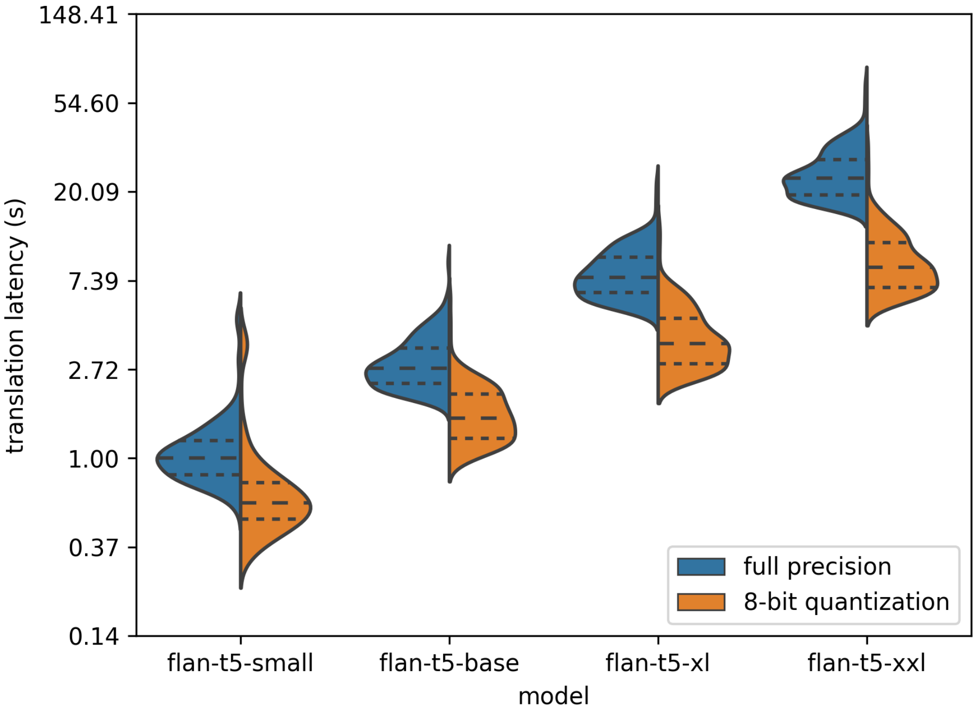

Figure S1: Distribution of translation latency with varying base models and quantization settings. Note that the y-axis is in the log scale.
